# Genomic exploration of *Bacillus paralicheniformis* TB197: an agrobiotechnological tool from the Sonoran Desert

**DOI:** 10.1128/spectrum.00441-26

**Published:** 2026-07-22

**Authors:** Estefany Chavarria-Quicaño, Miguel A. Hernández-Oñate, Jorge D. Payan-Almanza, Viridiana Rubio-Maldonado, Francisco de la Torre-González, Ali Asaff-Torres

**Affiliations:** 1 Department of Investigation, Development and Innovation, Innovak Global, Chihuahua, México; 2 SECIHTI - Centro de Investigación en Alimentación y Desarrollo, Hermosillo, Sonora, México; 3 Department of Food Science, Centro de Investigación en Alimentación y Desarrollo, Hermosillo, México; University of the Philippines Los Baños, Laguna, the Philippines

**Keywords:** bacteria, genome mining, bioestimulant, biocontrol, stress tolerance

## Abstract

Climate change and the harmful effects of extensive agrochemical use for plant nutrition and pest control on soils, the environment, and human health are driving the search for sustainable alternatives that reduce their use while increasing plant resilience. In regenerative agriculture, microorganisms have become valuable tools, acting as biological control agents or biostimulants, such as plant growth-promoting rhizobacteria, and/or to enhance plant performance under abiotic stress. The genus *Bacillus* is well known for its versatile interactions with plants. Specifically, *Bacillus paralicheniformis* TB197 has demonstrated high efficacy in controlling phytopathogenic nematodes and adapting to diverse soil and crop conditions. Based on these traits, we explored the agricultural potential of this strain through genomic analysis and *in vitro* and *in vivo* assays. Gene analysis identified functions related to three main areas: (i) stress resistance and plant colonization, (ii) plant growth promotion, and (iii) phytopathogen control. The strain showed high tolerance to salinity and temperature, promoted plant growth, and exhibited strong antifungal activity. These findings highlight the potential of the TB197 strain as a promising candidate for developing next-generation bioinoculants.

**IMPORTANCE:**

The use of beneficial microorganisms is a pivotal strategy for mitigating the environmental impacts of intensive agriculture while preserving crop productivity. *Bacillus paralicheniformis* TB197 is a native desert soil bacterium with genetic traits associated with stress tolerance, plant growth promotion, and suppression of plant pathogens. In this study, we employed a multifaceted approach integrating genomic analysis and functional assays to demonstrate the strain’s multifunctional potential as an agricultural bioinoculant. The results of the study demonstrate that a singular bacterial strain can integrate multiple beneficial functions relevant to sustainable agriculture. This work contributes to the field of applied microbiology by expanding the understanding of how environmentally adapted bacteria can serve as biological alternatives to chemical inputs in agroecosystems.

## INTRODUCTION

Over the past 65 years, the global population has increased rapidly from approximately 3 billion to 8 billion, creating a significant need for a substantial rise in food production (1). This has primarily been achieved through a substantial increase in crop yields, driven mainly by the extensive use of agrochemicals, including fertilizers and pesticides (2). However, despite the strict regulatory procedures involved in developing synthetic agrochemicals, their substantial environmental impacts are well-documented. These include soil and water pollution, greenhouse gas (GHG) emissions, land degradation, the development of pest and disease resistance, and adverse effects on human health (3–5)

The negative impacts of using chemical pesticides have led to a shift in farming practices, with a growing preference for biological control agents (BCAs) in disease management (6). The strong potential of BCAs as an alternative or addition to chemical control has increased their popularity as a key part of integrated pest management (IPM) strategies worldwide (7). Using biological control within the IPM framework promotes healthy crops while reducing effects on human health and the environment (7). BCAs, which typically include bacteria, protozoans, fungi, and viruses, are naturally occurring. Although the chemical fertilizer industry is considered highly polluting on its own, responsible for an estimated 5% of global heat-trapping emissions due to its large energy consumption, mainly from power plants that burn fossil fuels and emit carbon dioxide (CO_2_), about two-thirds of the industry’s emissions happen after farmers apply fertilizer in the field (8). This is because naturally occurring soil microbes convert excess fertilizer into various compounds, including N_2_O, which has a warming effect 265 times that of CO_2_ (9).

These concerns are driving significant changes in agricultural practices, which are being integrated into holistic systems, such as regenerative agriculture. This approach focuses on reducing the use of agrochemicals and increasing the use of natural fertilizers and beneficial microorganisms, such as plant growth-promoting rhizobacteria (PGPR) and others (10). Although the exact mechanisms by which PGPRs promote plant growth are not fully understood, some possible explanations include improving soil structure and remediating polluted soils by sequestering toxic heavy metals and degrading xenobiotic compounds; enhancing resistance to abiotic stress; biological nitrogen fixation (BNF); producing various plant growth regulators like abscisic acid (ABA), gibberellic acid (GA), cytokinins (CK), and auxin (indole-3-acetic acid, IAA); solubilizing and mineralizing nutrients, especially mineral phosphate; protecting plants from phytopathogens through mechanisms, such as antibiotic production, siderophore production, induction of systemic resistance, chelating available Fe in the rhizosphere, synthesizing extracellular enzymes to break down fungal cell walls, competing for niches in the rhizosphere; and reducing ethylene levels in developing plant roots by producing 1-aminocyclopropane-1-carboxylate (ACC) deaminase (11; 12). The main group of PGPRs includes genera, such as *Frankia*, *Acinetobacter*, *Arthrobacter*, *Azotobacter*, *Azospirillum*, *Streptomyces* spp., *Bacillus*, *Enterobacter*, *Burkholderia*, *Bradyrhizobium*, *Rhizobium*, *Serratia*, *Thiobacillus*, and *Pseudomonas* (13). The beneficial effects of PGPRs on plants have been well documented.

Since the term “PGPR” was introduced in the late 1970s (14), research teams around the world have studied the mechanisms, modes of action, physiology, biology, and chemistry of these organisms through traditional biochemical and genetic experiments known as “top-down” strategies (15). These approaches detect biological activities only under specific culture conditions and therefore reveal just a small part of the bacteria’s metabolic capabilities (12). However, the arrival of the genomic era has led to the development of new approaches and research methods for studying PGPR, resulting in a significant increase in available data that can be analyzed using genome mining, which involves examining whole-genome sequencing data to discover new enzymes, targets, natural products, or even intrinsic biological properties of a strain (16).

Since the active principles of PGPR are mainly mediated by enzymes and secondary metabolites, genome mining provides an opportunity to thoroughly analyze a PGPR strain’s whole genome for genes encoding beneficial enzymes involved in resource acquisition, phytohormone production, and regulation of plant hormone levels. It also allows for the identification of biosynthetic gene clusters (BGCs) that produce antimicrobial compounds (17). Additionally, other biological properties of PGPR, such as root colonization ability, the capacity to survive harsh conditions like high temperatures, drought, and salinity, and strain-specific traits, can be predicted using a genome-mining approach. Notably, many genes remain inactive under standard laboratory conditions due to a lack of appropriate natural triggers or stress signals, and some PGPR functions may be overlooked when relying solely on classical experimental methods (18). Whole-genome analyses offer the advantage of revealing and exploring the full spectrum of PGPR capabilities. Moreover, for future field applications, the safety and potential virulence of a PGPR strain can be more thoroughly assessed as the strain’s full capabilities are uncovered (16).

A key challenge facing agriculture in the 21st century is producing enough food sustainably to meet the needs of a rapidly growing population (2). This requires, among other actions, incorporating new and effective tools, such as PGPR, into agricultural practices. Previous studies from our group demonstrated that *Bacillus paralicheniformis* TB197, a native strain from the Sonoran Desert, exhibits strong nematicidal activity and the ability to colonize diverse crop environments (19), but its potential as a PGPR had not been studied. The present study aims to explore its broader agricultural potential employing genome mining to identify genes associated with plant growth promotion and pathogen control. *In vitro* and *in vivo* assays confirmed strong activities, positioning the TB 197 strain as a multifunctional beneficial microorganism beyond its nematicidal capability.

## MATERIALS AND METHODS

### Materials and reagents

Luria Bertani (LB) broth, Potato Dextrose Agar (PDA), and Yeast Extract Mannitol (YEM) were supplied by Becton Dickinson (San Miguel, Cuautitlán Izcalli, Estado de Mexico, Mexico). Carboxymethylcellulose Agar (CMC), Tryptone Soy Broth (TSB), Murashige and Skoog salts (MS), and Phytagel were provided by Sigma-Aldrich (Merck, Darmstadt, Germany). FeCl_3_, Salwowski reagent, NaCl, and KOH were supplied by HYCEL (Zapopan, Jalisco, Mexico).

### Bioinformatic analysis

The resulting contigs from the *de novo* genome assembly were polished and submitted to the National Center for Biotechnology Information (NCBI) GenBank as a whole-genome shotgun (WGS) under the BioSample and BioProject accession numbers SAMN48713388 and PRJNA1267181, respectively. Furthermore, annotation and gene prediction of TB197 were performed using the Prokaryotic Genome Annotation System (Prokka, v1.14.6) (20) and the NCBI Prokaryotic Genome Annotation Pipeline (PGAP) (21). The genome sequence of the TB197 strain was also mined to identify genes related to stress resistance and tolerance, colonization, and plant growth promotion using the Rapid Annotation Using Subsystem Technology (RAST) server version 2.0 (https://rast.nmpdr.org/rast.cgi?page=JobDetails&job=1477766) (22). Biosynthetic gene clusters associated with secondary metabolites with biological activities, including NRPS, PKs, NRPS-PKs (hybrid), RiPPs, bacteriocins, terpenes, and siderophores, were mined using the Antibiotic and Secondary Metabolite Analysis Accessory (AntiSMASH) online tool (https://antismash.secondarymetabolites.org) (23) and BAGEL4, a user-friendly web server for comprehensive mining of RiPPs and bacteriocins (http://bagel4.molgenrug.nl/) (24). Finally, genes encoding carbohydrate-active enzymes (CAZymes) in the TB197 genome were identified using the dbCAN3 database (https://bcb.unl.edu/dbCAN2) (25).

### Stress resistance and tolerance assays

To evaluate temperature tolerance, a single loop of TB197 or ATCC 21332 was inoculated into 50 mL of LB medium in 250-mL Erlenmeyer flasks, achieving initial cell counts of 1.7 × 10^5^ (± 3.1 × 10^4^) cells/mL (Neubauer chamber) and incubated in a shaker incubator (Thermo Scientific, Max*Q* 4000) at 180 rpm and 35°C, 45°C, 55°C, and 60°C for 48 h. For salinity tolerance testing, LB medium was supplemented with sodium chloride to 2%, 4%, 6%, 8%, and 10% (w/v). A single loop of the *B. paralicheniformis* TB197 strain was inoculated into 50 mL of each saline medium and incubated under the same conditions (180 rpm, 35°C) for 48 h (26).

In both assays, cell biomass concentration was measured based on total dry weight. Briefly, 50 mL of fermented broth was centrifuged at 10,034 × *g* for 30 min (Eppendorf 5804R). The pellet was washed, resuspended in distilled water, and centrifuged again under the same conditions. The resulting pellet was transferred to a pre-weighed dish and dried at 80°C until a constant weight was reached. Cell mass concentration was expressed as g/L.

### Antimicrobial activity assays

The *in vitro* antifungal antagonism assay was performed using a dual culture assay on PDA medium with slight modifications (27). A 7-day-old agar bud (0.5 cm in diameter) of *Fusarium oxysporum* or *Colletotrichum acutatum* was placed in the center of a PDA Petri dish. Then, four fungal buds from the potential BCAs *B. paralicheniformis* TB197 strain or *B. subtilis* ATCC 21332 strain were arranged crosswise about 2.5 cm around the fungus. After 6 days of incubation at 30°C in the dark, the width of the inhibition zone between the pathogen and the BCA was measured. Plates inoculated only with fungi served as controls. The following formula was used to calculate the antifungal activity of the bacteria isolated in the dual culture assay:


$$\%\;Inhibition=\frac{CD-TD}{CD}\times 100\%$$

Where CD: control phytopathogen diameter (cm); TD: treatment phytopathogen diameter (cm).

The relative antibacterial activity (%) effect on bacteria was tested using the well-diffusion assay, as described by Tejero-Sariñena et al. (28). A volume of 200 µL (0.200; OD600) of the plant-pathogenic bacteria *Erwinia amylovora* or *Xanthomonas campestris* was evenly spread on nutrient agar Petri dishes. Then, a 5 mm-thick disk was placed in the center of each plate and inoculated with 10 µL (1 × 10^7^ CFU/mL) of the potential BCAs *B. paralicheniformis* TB197 strain or *B. subtilis* ATCC 21332 strain (positive control, PC). The plates were incubated at 28°C for 48 h. The antibacterial activity was determined by measuring the inhibition halos and calculated using the following formula:


$$\%\;Relative\;antibacterial\;activity=\frac{PC-TD}{PC}\times 100\%$$

Where PC: positive control diameter (cm) and TD: treatment diameter (cm)

### Plant growth-promoting *in vitro* assays

#### Plant material and growth conditions

*Arabidopsis thaliana* accession Col-0 is the wild-type genotype. After 2 days of stratification at 4°C in the dark, *Arabidopsis* seeds were surface-sterilized with 20% (v/v) NaClO solution for 1 min. The seeds were germinated and grown on MS solid media plates containing Murashige and Skoog Basal Salts mixture (0.25× MS salts, 0.05% 2-(N-Morpholino) ethanesulfonic acid, 0.6% sucrose, 0.4% Phytagel; pH 4 adjusted with 1 M KOH) in square Petri dishes (10 × 10 cm^2^). Plants were vertically placed in a plant growth chamber (Percival Scientific, E-36VL) under a long-day photoperiod (16 h light/8 h dark) with a light intensity of 100 μmol m^2^/s at 22°C (29). After 4 days of growth, the seedlings were used in further experiments.

Seeds of romaine lettuce (*Lactuca sativa* var. longifolia) were grown under controlled conditions in a growth chamber (Percival AR75L3X). Environmental parameters were set to a 14-h light/10-h dark photoperiod, a constant temperature of 24°C, a light intensity of 200 μmol m^2^/s, and a relative humidity of 50%–60%. For germination, 50 seeds were planted in 72-cell trays filled with sterilized Berger BM2 substrate (a mixture of peat and perlite) and moistened with sterile water (30). After 10 days of germination, the seedlings were used in further experiments.

#### Colonization of roots

Root colonization was qualitatively assessed in *A. thaliana* seedlings grown in square Petri dishes containing MS solid medium, as described by Li et al. (29). One day before the experiment, the *Bacillus* strains (TB197 and ATCC 21332) were cultured overnight in Luria-Bertani (LB) liquid medium at 30°C and 180 rpm (Thermo Scientific, MaxQ 4000). Bacterial cells were washed twice with sterile saline solution (0.9% NaCl) and then centrifuged for 10 min at 4,300 × *g* and 4°C (OHAUS, FC5816R) to remove residual medium. The resulting pellets were resuspended in 0.01 M MgSO₄ solution to reach a final concentration of 10⁵ CFU/mL. A 5-μL aliquot of the bacterial suspension was placed 0.5 cm below the base of each 3-day-old seedling. Plates were sealed with Parafilm and incubated for 8 days in a growth chamber at 22°C. At the end of incubation, root colonization by the bacteria was observed using a Motic AE 20,000 inverted microscope (20×).

#### Evaluation of volatile organic compounds

For experiments involving bacterial volatile organic compounds (VOCs), bi-compartmented 9 cm-diameter Petri dishes were used. One compartment was filled with LB agar medium, while the other was filled with MS salts medium as described previously. One day before plant experiments, *Bacillus* strains (TB197 and ATCC 21332) were grown in Luria-Bertani (LB) liquid medium at 30°C and 180 rpm overnight (Thermo Scientific, MaxQ 4000). The bacterial cells were washed twice with 0.9% NaCl by centrifugation (OHAUS, FC5816R) for 10 min at 4°C and 4,300 × g and were finally resuspended in 0.01 M MgSO₄ to a concentration of 10⁵ CFU/mL. Four drops of 10 μL bacterial suspension (or distilled water as the control) were spotted on the side of the bi-compartmented Petri dishes containing LB agar. Three-day-old seedlings (four plants) were transferred to the other compartment containing MS salts solid media. The plates were sealed with Parafilm and incubated for 8 days in a growth chamber at 22°C. At the end of this period, primary root length and lateral root number were recorded (29) using a Canon EOS 5D camera with a 50-mm lens. Images were taken against a matte black, non-reflective background. The images were then analyzed using the digital image analysis software ImageJ v3.16.

#### Evaluation of the plant growth-promoting effect of *Bacillus* in lettuce plants

*Bacillus paralicheniformis* and *B. subtilis* were cultured in Luria-Bertani (LB) broth at 30°C for 24 h in an orbital shaker at 180 rpm (Thermo Scientific, MaxQ 4000). The bacterial cells were washed twice with sterile saline solution (0.9% NaCl), then centrifuged for 5 minutes at 4°C and 3,260 × *g* (Thermo Scientific, ST8). The cell pellets were resuspended in a 0.1% Tween 20 solution and adjusted to an optical density (OD_600_) of 2 using a spectrophotometer. A volume of 2 mL of each bacterial suspension (TB197 and ATCC 21332) was applied to eight lettuce plants at 10 days post-germination. A second application was made at 20 days. After 30 days of growth, several plant growth-promoting parameters were measured, including the number of leaves (counted manually), dry biomass (shoots and roots, dried at 80°C for 24 h), and leaf area (31).

For leaf area measurement, lettuce leaves were carefully detached from the plants and photographed together using a Canon EOS 5D camera with a 50-mm lens. Images were captured on a matte black, non-reflective background. The images were then analyzed with the digital image analysis software ImageJ v3.16. Spatial calibration was performed using the software’s scale tool, based on a metric reference (cm/pixel). Next, the “Color Threshold” tool was applied, with manual adjustments to the color channels in HSB mode, to isolate the leaf tissue and exclude the background and other non-target elements. Finally, the “Analyze > Measure” function was used to determine the projected area of each leaf, expressed in square centimeters (cm²) (32).

### Siderophores, cellulases assays, and IAA assays

A standard colorimetric assay was used to measure IAA production in the culture broth (33). The isolates were inoculated into 50 mL of sterilized Yeast Extract Mannitol (YEM) broth supplemented with 0.1 g/L of L-tryptophan in 250-mL Erlenmeyer flasks, and incubated in a rotary shaker incubator (Thermo Scientific, MaxQ 4000) at 100 rpm and 28°C for 4 days. After incubation, the culture broth was centrifuged (OHAUS, FC5816R) at 3,000 × *g* for 3 min, and the supernatant was mixed with an equal volume of Salkowski reagent (1 mL of 0.5 M FeCl_3_ in 50 mL of 35% HClO_4_). Following a 30-min incubation in the dark, absorbance was measured at 530 nm, and IAA concentrations in each sample were determined using a calibration curve with known IAA standards (10–100 μg/mL). As a negative control, 2 mL of YEM broth was mixed with an equal volume of Salkowski reagent. The experiments included three biological replicates and three technical replicates.

The semiquantitative assay for siderophore production was conducted using the chromium azurol sulfonate (CAS) agar plate method, as described by Schwyn and Neilands (1987) (34) with modifications as described by Barcos-Arias et al. (35). The tested strains were cultured on LB medium for 48 h using a rotary shaker at 180 rpm and 30°C. Then, 10 µL of a bacterial suspension containing approximately 5 × 10^7^ cells/mL was placed in the center of CAS agar plates and incubated for 5 days at 30°C. An orange halo around the bacteria on CAS blue agar indicates the strain’s ability to produce siderophores. The siderophore production index (SPI) was calculated using the following formula:


$$SPI=\frac{D_{h}-D_{c}}{D_{c}}$$

Where total diameter of the halo (including the colony) in cm; diameter of the bacterial colony (cm).

Semiquantitative cellulase activity was assessed using carboxymethylcellulose (CMC) agar plates and Gram’s iodine staining, as described by Chantarasiri et al. (36). A 5-µL sample of overnight culture broth (Tryptone Soy Broth) from TB197 or ATCC 21332 strains was placed at the center of CMC agar plates containing 0.2% NaNO_3_, 0.1% K_2_HPO_4_, 0.05% MgSO_4_, 0.05% KCl, 0.2% CMC, 0.02% peptone, and 1.7% agar. The plates were incubated at 28°C for 48 h. Afterward, they were immersed in Gram’s iodine solution for 10 min. Cellulolytic activity was indicated by the formation of a hydrolytic halo around colonies after iodine staining. The following formula was used to determine the hydrolytic capacity:


$$HI=\frac{TD}{CD}$$

Where HI: hydrolysis index; TD: total diameter of the halo (including the colony, cm); CD: diameter of the bacterial colony (cm).

### Statistical analysis

All experiments were conducted using a completely randomized design (CRD) and analyzed with one-way ANOVA. Data normality was assessed using the Shapiro–Wilk test at the *P* < 0.05 significance level. Differences among means in stress tolerance, siderophore production, cellulolytic activity, IAA synthesis, and antimicrobial activity assays were evaluated using Student’s *t*-test. For leaf number, dry weight, and leaf area, mean comparisons were performed with the Tukey–Kramer test, only when ANOVA indicated significant effects. Results are reported as mean ± standard deviation (SD). All statistical analyses were conducted using NCSS v11 (NCSS, LLC, Kaysville, Utah, USA), and all graphs were created with GraphPad Prism v8.0 (GraphPad Software, San Diego, CA, USA).

The stress tolerance assays, cellulase activity, siderophore production, and antifungal and antibacterial activity tests of *Bacillus* strains (TB197 and ATCC 21332) were conducted using a design with three biological replicates, including three technical replicates each. For the plant growth promotion assays, two biological replicates were performed, each with 14 technical replicates. In the volatile organic compound (VOC) exposure and root colonization experiments, five plants per treatment were used. In the lettuce (*Lactuca sativa*) assays, 14 plants were used per treatment.

## RESULTS AND DISCUSSION

**Fig 1:**
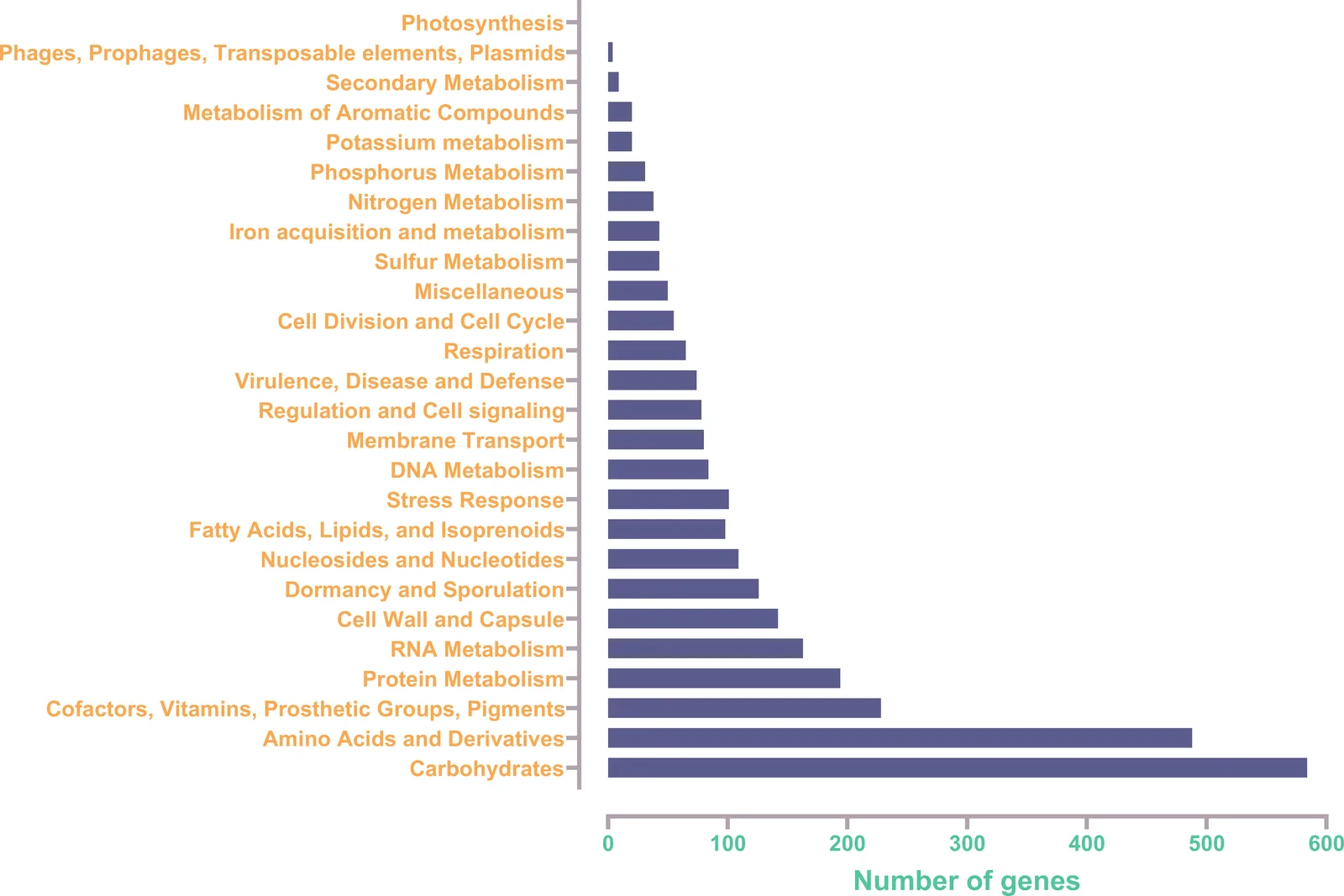
Functional annotation of *Bacillus paralicheniformis* TB197 strain: distribution of subsystems by RAST.

Bacteria of the genus *Bacillus* are ubiquitous in the soil and are often abundant in the plant rhizobiome, where they promote plant growth and protect against both abiotic and biotic stresses (37, 38). Their beneficial effects on plants have been extensively studied by multiple research groups (39, 40). In this study, we thoroughly evaluated the agricultural potential of the *Bacillus paralicheniformis* TB197 strain. Genome functional annotation was carried out using RAST (Fig. 1). The figure illustrates the distribution of genes across various functional categories, which are explained in detail below.

### Elements involved in stress resistance, soil, and plant colonization

#### Stress resistance and tolerance

**Fig 2:**
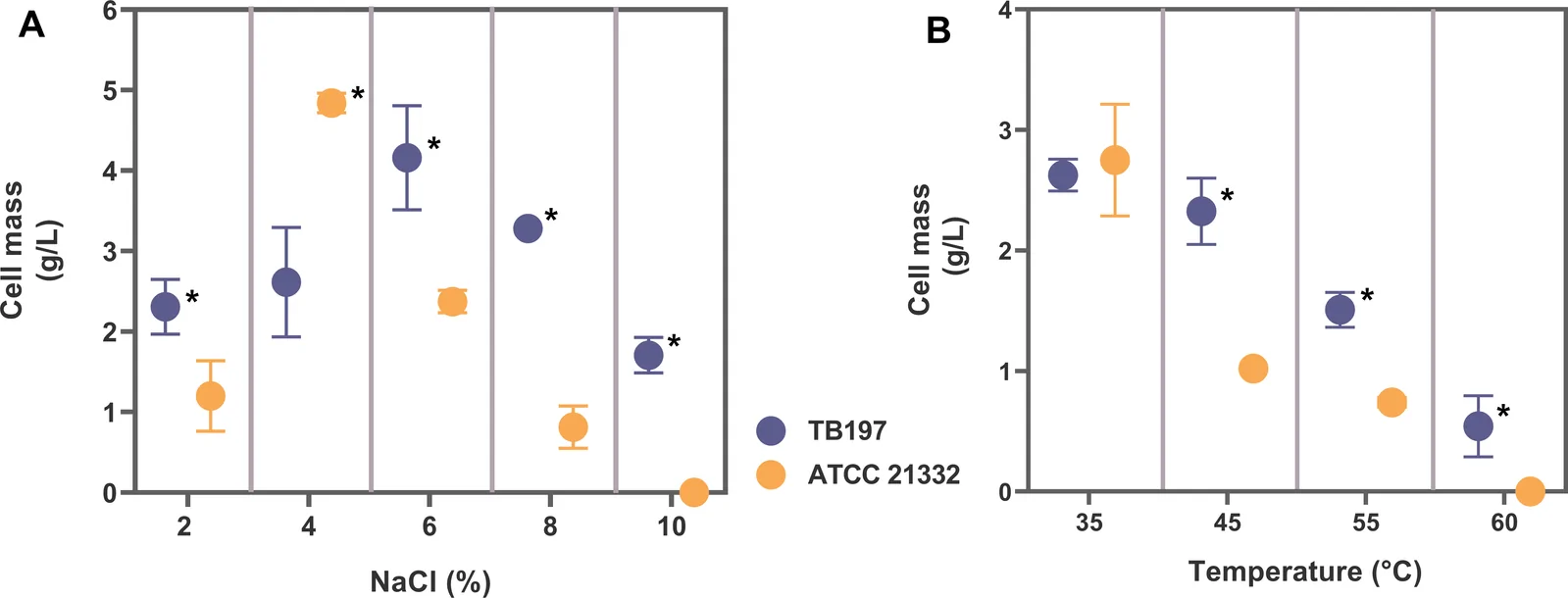
Effect of saline stress (**A**) and thermal stress (**B**) on the growth of *Bacillus paralicheniformis* TB197 and *Bacillus subtilis* ATCC21332. Data are shown as mean ± SD. Statistical differences between *Bacillus* strains within each condition were evaluated using Student’s *t*-test. Asterisks (*) indicate significant differences *P* < 0.05, relative to the corresponding group.

Genome analysis of the *B. paralicheniformis* TB197 strain revealed several genes involved in osmotic and salt stress tolerance, including sodium and chloride transporters, the osmoprotectant transport system, osmolyte biosynthesis and transport (e.g., glycine-betaine, proline, glutamate, glutamine), and membrane integrity and protection mechanisms (e.g., cardiolipin, lipoteichoic acid production, fatty acid desaturases) (Functional annotation data are available in figshare: 10.6084/m9.figshare.29213153). The abundance of genetic elements related to osmotic and salt stress tolerance in the TB197 genome aligns with the *in vitro* analysis results (Fig. 2A), which showed increased growth at elevated NaCl concentrations, even at 10% NaCl, where the reference *Bacillus subtilis* ATCC 21332 was unable to grow. Although NaCl generally inhibits rather than enhances bacterial growth by creating high osmotic pressure, the dose-response curves for both *B. paralicheniformis* and *B. subtilis* exhibit Gaussian behavior, consistent with other reports that some soil bacteria may show increased growth as salt concentrations rise until an optimal point (41, 42).

Our findings on the osmotic and salt tolerance of the TB197 strain align with those of previous reports. For example, Ibarra-Villarreal et al. (43) found that *B. paralicheniformis* TRQ65 can grow in the presence of 5% reported that the strain *B. paralicheniformis* ES-1, isolated from a salt mine in Karak, Pakistan, could grow in up to 9.8% NaCl, which matches the tolerance levels observed in this study (34). Notably showed that *B. paralicheniformis* BL-1 not only tolerates high salinity (10% NaCl) but also maintains its ability to degrade phenol under these extreme conditions (42). These findings emphasize the remarkable halotolerance and metabolic versatility of *B. paralicheniformis* strains, supporting their potential for biotechnological applications in saline environments.

The study of microbial strains that tolerate salt stress is particularly important because soil salinization is a widespread environmental problem that restricts agriculture and threatens food security, affecting approximately 954 million hectares worldwide (44). Salt stress, mainly caused by sodium chloride (NaCl), disrupts ionic and osmotic balance in plants, leading to ionic toxicity, nutritional deficiencies, and the production of reactive oxygen species (ROS) (45). These ROS can cause oxidative damage and lead to cell death. In this context, salt-tolerant plant growth-promoting rhizobacteria (ST-PGPR) are considered effective biotechnological tools (46). Their ability to tolerate salt allows them to thrive and colonize the rhizosphere in saline soils, helping plants cope with salt stress through direct mechanisms, such as producing phytohormones and enhancing nutrient availability, and indirect mechanisms, such as activating antioxidant and osmoregulatory defenses. These processes help mitigate the physiological effects of salt stress on crops (44, 46).

Thermal stress-related genes were also identified in the TB197 strain and were classified into at least five functional groups (Functional annotation data are available in figshare; 10.6084/m9.figshare.29213153). Notably, the chaperones *Dna*K, *Dna*J, and *Grp*E are involved in maintaining the stability of denatured proteins (47). Transcription factors Sigma32 and *Hrc*A regulate the expression of heat response genes (48). Enzymes from the SAM family and various methyltransferases contribute to the stabilization of RNA and ribosomes (49). To verify the thermal tolerance of the TB197 strain, its growth was tested across a range of temperatures (Fig. 2B). The TB197 strain grew at temperatures up to 60°C, whereas the reference ATCC 21332 strain grew only up to 50°C. This ability allows us to classify the TB197 strain as a thermophilic bacterium.

The search for heat-stress-tolerant microbial strains has become increasingly important due to global warming and rising temperatures, which have led to crop yield losses of 15%–35% in regions, such as Africa and Asia (50). Similar to salt stress tolerance, thermophilic bacteria can thrive and colonize rhizospheric environments where soil temperatures exceed 45°C (51), a temperature range where mesophiles cannot survive. Several studies indicate that heat-tolerant plant growth-promoting rhizobacteria can enhance thermotolerance in crops (52). Specifically, PGPR strains aid by producing phytohormones, forming biofilms, reducing abscisic acid (ABA) levels, and increasing heat shock protein (HSP) production (50). These findings emphasize the importance of genomic and physiological analyses of strains such as TB197 for potential agricultural applications under high-temperature conditions.

#### Spore formation

Several genes and enzymes that regulate spore formation and dormancy are widely distributed in the TB197 genome (Functional annotation data are available in figshare: 10.6084/m9.figshare.29213153). These include genes involved in dipicolinate synthesis and the production of small acid-soluble spore proteins (SASPs), which are vital for protecting the spore core. Additionally, a range of genes belonging to the Sporulation Cluster IIIA, spore germination pathways, and the SpoVS protein family were identified, along with numerous sporulation orphan genes. In previous studies, we reported that strain TB197 can develop sporulating phenotypes, supporting the identification of genes involved in sporulation (53). The ability to form spores has been demonstrated to improve the resilience of *Bacillus* strains to environmental stresses, including drought, nutrient shortages, and other ecological challenges. This trait is advantageous for developing biotechnological products, as it simplifies their formulation, storage, and handling, particularly for agricultural and industrial applications (38).

#### Heavy metal resistance

Some bacteria in the genus *Bacillus* have diverse genetic systems that provide resistance to environments contaminated with heavy metals and metalloids. In particular, several genes linked to heavy metal resistance were found in strain TB197, including *cop*A, *cop*Z, *cso*R_1, and *cso*R_2 (54), which are involved in expulsion, transport, and regulation against high copper levels (Functional annotation data are available in figshare: 10.6084/m9.figshare.29213153). Additionally, the *czc*D gene encodes an antiporter that expels Cd²^+^, Co²^+^, and Zn²^+^ ions by exchanging them with H^+^ or K^+^, helping maintain cellular ionic balance (55). Conversely, the *ars*C and *arc*B genes are associated with arsenic resistance, working through mechanisms that include reducing arsenate to arsenite and expelling it from the cell, which mitigates the metalloid’s toxicity (56).

The presence of heavy metal resistance genes in PGPR provides a key adaptive advantage, enabling them to survive and effectively colonize contaminated soils (55). Numerous studies have shown that species belonging to the *Bacillus* genus not only actively participate in bioremediation through mechanisms such as biosorption, precipitation, and redox transformation of metals, but also help protect plants by reducing oxidative stress (57–59). This activity has been proven to improve plant health, growth, and development in challenging environments (60).

#### Motility, chemotaxis, and attachment of plant surfaces

Bacilli are motile, facilitated by flagella, which enable them to navigate toward nutrient sources and attach to host plants. The coordinated expression of multiple structural, motor, regulatory, and chemotaxis genes is a key aspect of bacterial motility. Genome analysis of the TB197 strain revealed genes involved in flagella synthesis and assembly, including *hag*, *flg*E, *flg*K, and *flg*L (Functional annotation data are available in figshare: 10.6084/m9.figshare.29213153), which are responsible for filament and hook formation. The *mot*A and *mot*B genes, which are necessary for flagellar motor function, were also identified. Additionally, chemotaxis-related genes, such as *che*A, *che*Y, *che*W, *che*B, *che*R, *che*V, *che*C, *che*X, and *che*Z, which are involved in detecting and processing environmental chemical signals, were found. These signals are detected by a family of MCP (methyl-accepting chemotaxis proteins) receptor genes, including *tsr*, *tar*, *trg*, *tap*, and *aer*, which allow bacteria to adjust their flagellar movement toward nutrient sources or away from toxic substances.

The process of biofilm formation in members of the *Bacillus* genus is highly regulated and depends on the coordinated expression of several key genes. In the genome of strain TB197, a set of genes putatively associated with biofilm formation was identified through *in silico* analysis. Among these, the *tas*A gene was identified as encoding an essential protein involved in biofilm formation. This protein is a major structural component of the extracellular matrix, providing stability and cohesion to the biofilm. The precise secretion and assembly of this protein complex rely on the actions of *yqx*M and *sip*W. Additionally, the metalloprotease Camelysin (*cam*) and the CalY/TasA family proteins Prot1, Prot2, and Prot3 have also been implicated in biofilm formation and stabilization (see Functional annotation data are available in figshare: 10.6084/m9.figshare.29213153).

**Fig 3:**
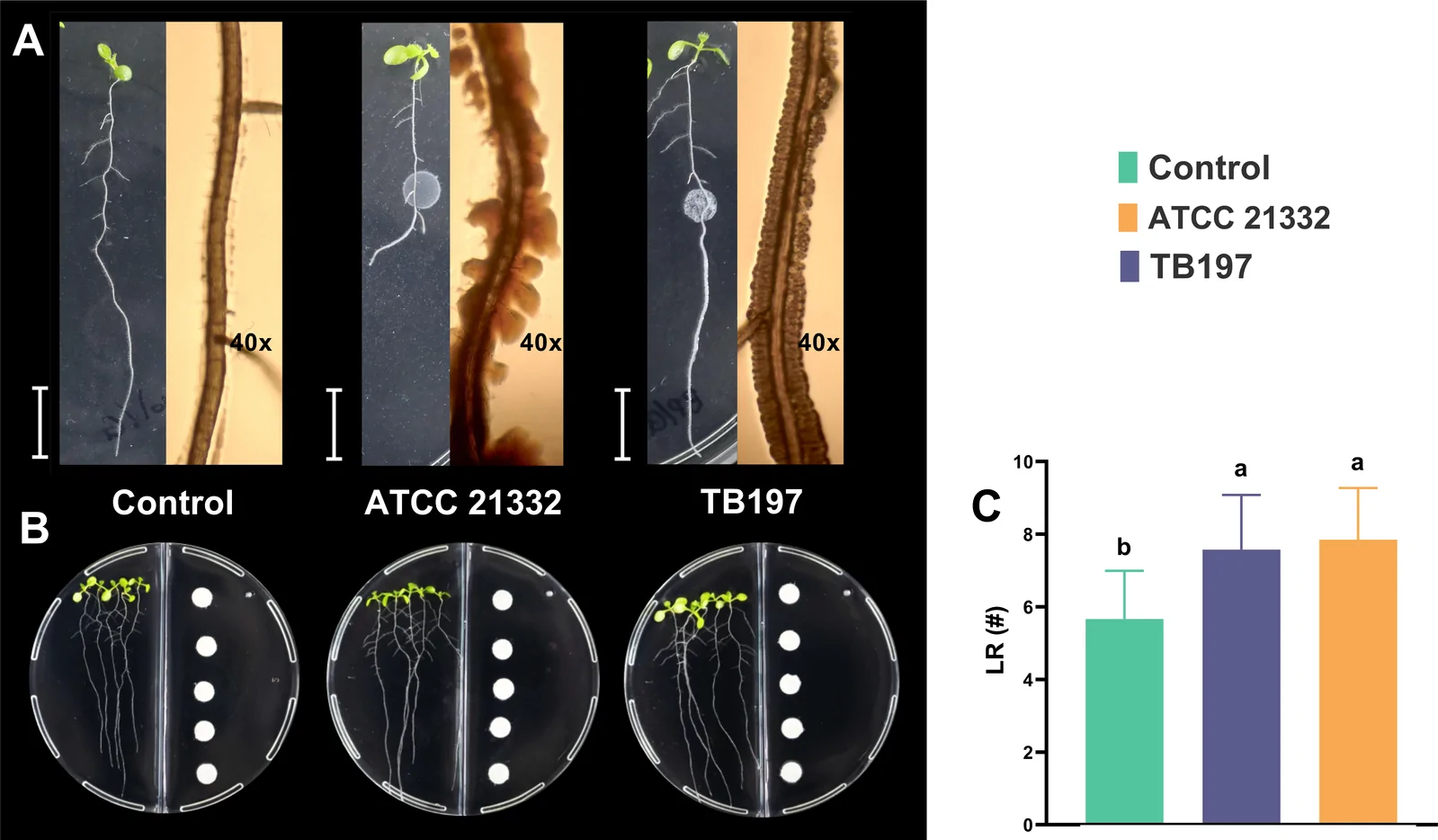
Root colonization and the effect of volatile organic compounds by *Bacillus* species. (**A**) Root colonization: on the left side of each panel, *Arabidopsis thaliana* seedlings grown in petri dishes (white lines indicate scale bar = 2 cm); on the right side, corresponding roots observed under a microscope at 40× magnification (images shown for qualitative comparison of bacterial root colonization). (**B and C**) Impact of bacterial volatile organic compounds (VOCs) on lateral root formation in *Arabidopsis thaliana*. Different letters indicate statistically significant differences between treatments (Tukey-Kramer, *P* < 0.05).

The ability to move and adhere to plant surfaces is crucial for effective colonization of roots by rhizospheric bacteria. In previous studies, the capacity of strain TB197 to form biofilms and exhibit motility in liquid culture was assessed (53), and the expression of key genes involved in these processes was observed. Similarly, in this work, the strain’s ability to colonize *A. thaliana* roots was tested in an *in vitro* comparative study with the well-known PGPR ATCC 21332 strain. The results showed that both bacterial strains exhibited strong adhesion to the root surface (Fig. 3A), confirming that TB197 is a vigorous colonizer of the root.

### Elements involved in plant growth promotion activities

#### Volatile organic compounds

Species of the genus *Bacillus* synthesize diverse volatile organic compounds (VOCs) that, beyond their well-known antimicrobial effects, can directly modulate endogenous phytohormone synthesis in plants (61). In the genome of strain TB197, genes involved in the synthesis of acetoin and 2,3-butanediol (*als*S, *bud*C, and *bdh*A) were identified (Functional annotation data are available in figshare: 10.6084/m9.figshare.29213153); these compounds have been previously associated with promoting root growth (39). In this study, this effect was tested in *A. thaliana*, where plants inoculated with strains TB197 and ATCC 21332 developed 1.36 times more lateral roots (*P* ≤ 0.05) compared with the uninoculated control (Fig. 3B and C). These findings support previous reports indicating that VOCs, particularly acetoin produced by *B. amyloliquefaciens*, stimulate lateral root formation through auxin-dependent mechanisms (29).

#### Phytohormone production

Genes involved in phytohormone biosynthesis were identified in the genome of strain TB197, including putative pathways for the production of indole-3-acetic acid (IAA) and γ-aminobutyric acid (GABA) (Functional annotation data are available in figshare: 10.6084/m9.figshare.29213153). IAA is a key auxin in plant–microbe interactions, regulating cell division, elongation, differentiation, apical dominance, and stress responses (62). IAA production was measured in supernatants from submerged cultures of TB197 and the reference strain ATCC 21332, yielding 10.03 and 4.88 mg/L, respectively, indicating that TB197 produced 2.1 times higher levels of IAA (*P* ≤ 0.05).

Although genes related to GABA biosynthesis were also found, its production was not experimentally quantified in this study. GABA has been linked to responses to biotic and abiotic stress, carbon–nitrogen balance, and the regulation of plant development (63).

**Fig 4:**
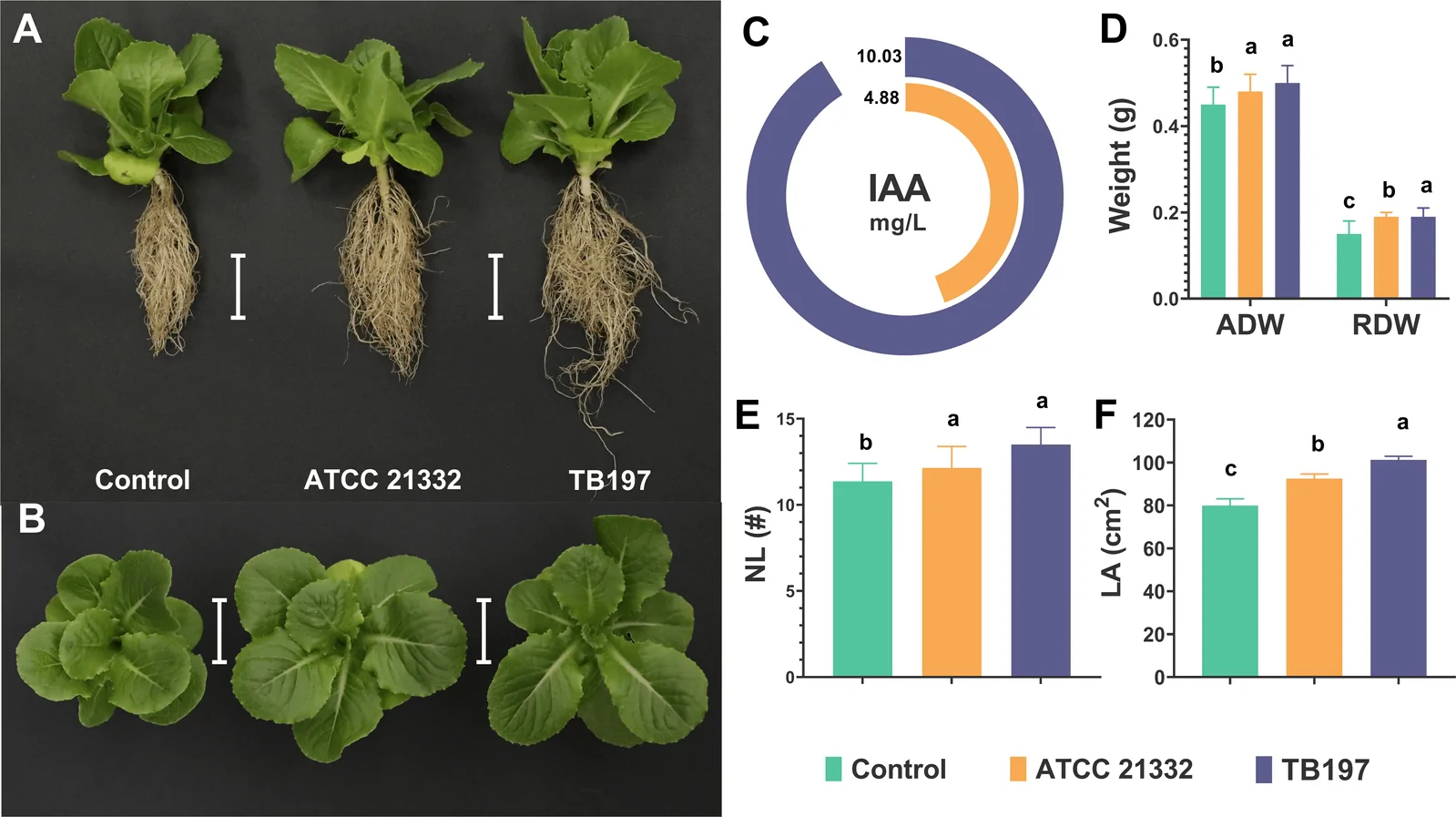
Plant growth-promoting effects of Bacillus strains. (**A and B**) Effect of inoculation with *Bacillus* strains ATCC 21332 and TB197 on lettuce growth under controlled conditions in a growth chamber (white lines indicate scale bar = 4 cm). (**C**) Indole-3-acetic acid production by Bacillus strains. (**D**) Leaf area of lettuce plants (cm²). (**E**) Biomass of lettuce plants; ADW: aerial dry weight; RDW: root dry weight. (**F**) Number of leaves of lettuce plants. Different letters indicate significant differences among treatments (*p* < 0.05).

To assess the functional effects of both strains on plants, bioassays were performed using lettuce seedlings under controlled conditions. Inoculation with TB197 and ATCC 21332 strains significantly enhanced several morphological parameters compared with the uninoculated control (Fig. 4A and B). First, aerial growth was improved, with the number of leaves increasing by 20% compared with uninoculated plants (*P* ≤ 0.05; Fig. 4C), and there was no difference between plants inoculated with ATCC 21332 and those inoculated with TB197. Leaf area reached 101.25 cm² (TB197), 92.50 cm² (ATCC 21332), and 79.94 cm² (uninoculated plants), representing increases of 40% and 20%, respectively, compared with the control (*P* ≤ 0.05; Fig. 4D and Fig. S1). Additionally, shoot dry weight increased by 30% in treated plants (with no difference between them) compared with the control (*P* ≤ 0.05; Fig. 4E). Furthermore, root development was improved (Fig. 4B), resulting in 40% greater root dry weight (RDW) in treated plants (no difference between them) compared with the control (*P* ≤ 0.05; Fig. 4E).

Overall, these results confirm that both strains act as plant growth-promoting rhizobacteria (PGPRs), with the *B. paralicheniformis* TB197 strain showing superior performance. This is likely due to its higher IAA production and VOCs emission, which enhance root development and improve nutrient uptake.

#### Nitrogen, sulfur, and phosphorus acquisition

Genes involved in nitrogen, sulfur, and phosphorus metabolism were identified in the genome of the TB197 strain (Functional annotation data are available in figshare: 10.6084/m9.figshare.29213153). Notably, several genes related to nitrogen metabolism were detected, which could help the strain tolerate nitrosative stress in both aerobic (e.g., *hmp, hcp, hcr*, *frd*X, *nsr*R, *nor*R, *hcp*R) and anaerobic (e.g., *nor*V, *nor*W, *qnor*, *dnr*N, *nor*R, *nnr*S, *hcp*R) environments (64). The presence of these genes suggests that the TB197 strain can survive in nitrogen-rich soils, especially those receiving high nitrogen fertilizer input (64).

Genes encoding nitric oxide synthases (NOs) were also identified, which is relevant because NOs production in certain rhizobacteria has been linked to symbiotic functions, such as regulating nodule formation in legume roots (65). Additionally, genes involved in nitrite and nitrate ammonification (*nir, nar*) were detected, along with key genes necessary for ammonium assimilation, including *gln*A, *glt*B, and *glt*D (66).

Importantly, genomic clusters encoding the main denitrification reductases (*nar*GHIJ, *nir*S/K, *nor*BC, *nos*Z) were also identified, which catalyze the step-by-step reduction of nitrate (NO₃⁻) to molecular nitrogen (N₂) (67). This process is important not only in nitrogen-rich soils, where it may help prevent nitrate toxicity, but also plays a key role in reducing nitrous oxide (N₂O) emissions, a potent greenhouse gas often produced in agricultural soils (65, 67). Further research is needed to understand how and when these genes are expressed in the TB197 strain, as well as their potential role in promoting plant growth under high-nitrogen conditions.

Similarly, genome analysis revealed a full spectrum of genes related to sulfur assimilation, including sulfate transport, its reduction to sulfide, and the incorporation of sulfide into sulfur-containing amino acids. The ABC transporter *cys*PUWA encodes a high-affinity SO₄²⁻ uptake system, while *sul*P and *yln*A are predicted secondary permeases based on homology and conserved domain analyses. The pathway for assimilatory sulfate reduction includes sat (ATP sulfurylase), *cys*D and *cys*N (APS reductase), *cys*C (PAPS reductase), *cys*H (sulfite reductase [hemoprotein]), *cys*J/I (sulfite reductase [flavoprotein]), and *cys*K/*cys*M (O-acetylserine [thiol]-lyase) (68). Additionally, the *ssu*EADCB and *tau*ABCD operons facilitate the uptake and breakdown of sulfonates and taurine through FMN-dependent monooxygenases (SsuD/SsuE) and the α-ketoglutarate-dependent dioxygenase TauD, releasing bioavailable sulfite. This metabolic capability demonstrates a high level of versatility in sulfur acquisition across various soil environments, including those with limited inorganic sulfate, potentially improving environmental adaptability and competitive advantage (69, 70).

Phosphate transport system genes, along with the alkaline phosphatase genes *pho*A and *pho*D, are present in the genome of the TB197 strain (Functional annotation data are available in figshare: 10.6084/m9.figshare.29213153), reflecting its main ability to acquire organic phosphorus (71).

#### Polyamine and modulation of ethylene levels

In the genome of the TB197 strain, the genes *spe*ABCEF and *paiA,* which are involved in spermidine biosynthesis, were identified (Functional annotation data are available in figshare: 10.6084/m9.figshare.29213153). Previous studies have demonstrated that polyamines can inhibit ethylene production, a hormone often referred to as the “stress hormone” in plants, which is activated under biotic and abiotic stress conditions (72). For instance, Nikhil et al. (73) reported that polyamines produced by *B. endophyticus* help reduce salt stress in *A. thaliana*.

Overall, these findings suggest that the TB197 strain possesses a strong genetic toolkit for promoting plant growth, and several of its beneficial traits have been confirmed *in vitro*. However, many functions still require further investigation, including testing this strain on stressed crops to elucidate the primary molecular mechanisms involved and how the bacterium mitigates stress in plants.

#### Iron acquisition

**Fig 5:**
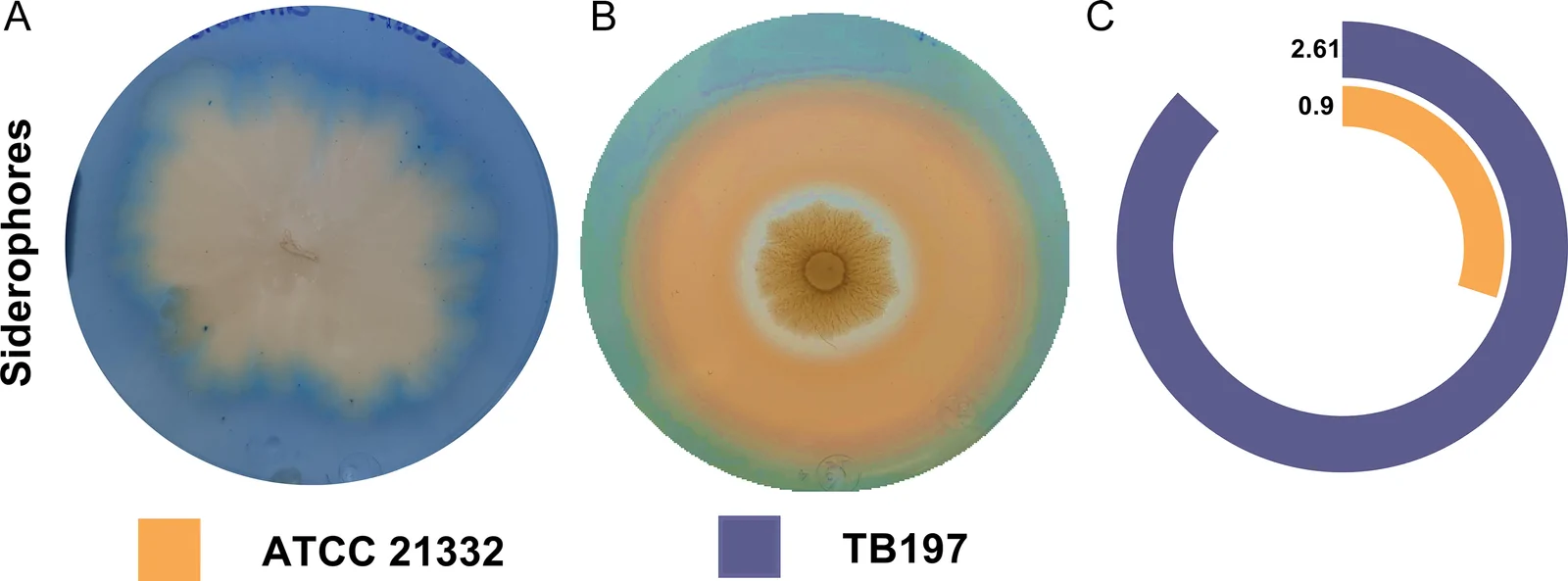
Semiquantitative production of siderophores. (**A**) *B. subtilis* ATCC 21332; (**B**) *B. paralicheniformis* TB197 strain; (**C**) siderophore production index.

Several genes linked to iron homeostasis have been identified in the genome of the TB197 strain. These include *hmo*A, *hmo*B, and the ferric uptake regulator (*fur*), which are involved in the uptake, transport, and regulation of intracellular iron. Additionally, analysis with antiSMASH identified three biosynthetic clusters associated with siderophore production. As shown in Table 1, the substances identified are schizokinen, pulcherriminic acid, and bacillibactins E/F. These compounds have a high affinity for ferric ions (Fe^3+^), indicating a strong potential for iron mobilization and uptake in environments with limited availability of this micronutrient. To experimentally confirm the genomic findings, a semiquantitative assay was performed on CAS agar. This assay compared siderophore production between the TB197 and ATCC21332 strains. The results (Fig. 5) demonstrate that TB197 produces 2.9 times more siderophores than the reference strain. These results support the bioinformatics predictions and suggest a significantly greater capacity for metabolite secretion.

**TABLE 1:** List of putative secondary metabolite-producing biosynthetic clusters as predicted by antiSMASH, Bagel4, and RAST^*a*^

	Clusters	Regions	Size (bp)	Most similar known biosynthetic cluster	Similarity (%)	Predicted by
1	NRPS	8.1	25,563	Surfactin	53	AntiSMASH
2	Thiopeptide LAP	17.1	41,204	Butirosin A/B	7	AntiSMASH
3	NI-siderophore	17.2	33,463	Schizokinen	60	AntiSMASH
4	NRPS	19.1	27,523	Fengycin	100	AntiSMASH
5	Terpene	22.1	21,889	Unknown		AntiSMASH
6	T3PKS	22.2	41,097	Unknown		AntiSMASH
7	NRSP	22.3	82,829	Bacitracin	100	AntiSMASH
8	CDPS	24.1	20,749	Pulcherriminic acid	66	AntiSMASH
9	NRP-metallophore	25.1	51,755	Bacillibactin E/F	100	AntiSMASH
10	RiPP		20,000	Lasso_peptide		Bagel 4
11	RiPP		20,000	Sactipeptide		Bagel 4
12	RiPP/bacteriocin		20,309	Sonorensin		Bagel 4
13	RiPP/bacteriocin		20,000	Bottromycin		Bagel 4
14	RiPP		20,000	Sactipeptide		Bagel 4
15	RiPP		20,000	Sactipeptide		Bagel 4
16	Competence		20,111	ComX, pheromone		Bagel 4
17	RiPP/bacteriocin		20,192	Enterosin Nkr-5-3B		Bagel 4
18	RiPP		20,000	Microcin modified by tiazol/oxazol (TOMM)		RAST

^*a*^ NRPS: non-ribosomal peptide synthetase; LAP: linear azole-containing peptides; NI: not clear structure; T3PKS: type III polyketide synthase; CDPS: cyclodipeptide synthetase; NRP-metallophore: non-ribosomal peptide-metallophore; RiPP: ribosomally synthesized and post-translationally modified peptides.

It is noteworthy that strain TB197 contains three separate biosynthetic clusters for siderophore production, each with different chemical and functional traits. The bacillibactins, for example, are catecholate-type siderophores with an extremely high affinity for ferric ions (Fe³^+^), making them highly effective at iron chelation, especially in acidic environments (74). In contrast, schizokinen belongs to the hydroxamate class, which has intermediate affinity for iron and functions better in neutral or alkaline conditions, thus expanding the strain’s ecological range of iron uptake (75). Finally, pulcherriminic acid, a derivative of a cyclodipeptide, forms pulcherrimine when it complexes with Fe³^+^. Pulcherrimine is an insoluble pigment that sequesters iron almost irreversibly. This compound plays a key ecological role by limiting iron availability to competing microorganisms, including phytopathogens (76). It has also been linked to the autoregulation of growth in *Bacillus subtilis*. Recent studies have shown that under iron-limited conditions, the accumulation of pulcherrimine can hinder the strain’s own biofilm development, prompting survival responses, such as sporulation (77).

The simultaneous production of these three types of siderophores by TB197 suggests a complex adaptive strategy that may enable it to regulate iron availability and support plant nutrition while effectively competing across diverse microbial environments. However, a deeper understanding of the molecular mechanisms that control the expression and activation of these genes under different environmental conditions remains crucial.

Siderophores are essential for iron uptake, forming soluble siderophore-Fe³^+^ complexes that plant roots can absorb. Once inside plant tissues, these complexes break down, reducing iron to Fe²^+^. This form of iron is primarily used by plants for processes such as chlorophyll synthesis and chloroplast maintenance (78). Additionally, the reduced availability of free iron in the rhizosphere may also limit the growth of plant pathogens, increasing the potential of TB197 as a plant growth-promoting and bioprotective agent.

### Elements involved in phytopathogenic control

#### Antibacterial activities

Approximately 13 genes involved in the biosynthesis of secondary metabolites with potential antibacterial activity were identified. These include non-ribosomal peptide synthetases (NRPSs), responsible for producing compounds such as surfactin and bacitracin; thiopeptide linear azole-containing peptides (LAPs), like butirosin; and several ribosomally synthesized and post-translationally modified peptides (RiPPs), including sonorensin, bottromycin, and enterocin Nkr-5-3b. Additional uncharacterized post-translationally modified peptides were also detected and classified as lasso peptides and sactipeptides, which may represent novel bioactive molecules that have not yet been described or experimentally validated (Table 1).

*In vitro* relative antibacterial assays showed that strain TB197 exhibited significantly lower activity compared with the reference strain ATCC 21332 (Fig. S2), being 1.00 and 3.74 times less effective against *Xanthomonas campestris* and *Erwinia amylovora*, respectively, two important agricultural phytopathogens. This was unexpected, given the abundance of biosynthetic gene clusters related to antibacterial compounds in the TB197 genome. One possible explanation is that the expression of these genes may depend on specific environmental cues, as they are not necessarily active under standard laboratory conditions (18). Additionally, among the detected metabolites, only surfactin showed activity against Gram-negative bacteria such as *E. amylovora* and *X. campestris*. In contrast, most bacteriocins (79), lasso peptides (80), and sactipeptides (81) described in the literature are primarily highly effective against Gram-positive bacteria.

Future work will focus on evaluating the antibacterial activity of the TB197 strain against Gram-positive plant pathogens (e.g., *Clavibacter michiganensis*, *Rhodococcus fascians,* etc.) and analyzing the associated metabolites. This approach will help improve understanding of the functional relevance of the identified biosynthetic gene clusters (BGCs) and may lead to the discovery of novel bioactive compounds or structural variations of known metabolites.

#### Antifungal activities

**Fig 6:**
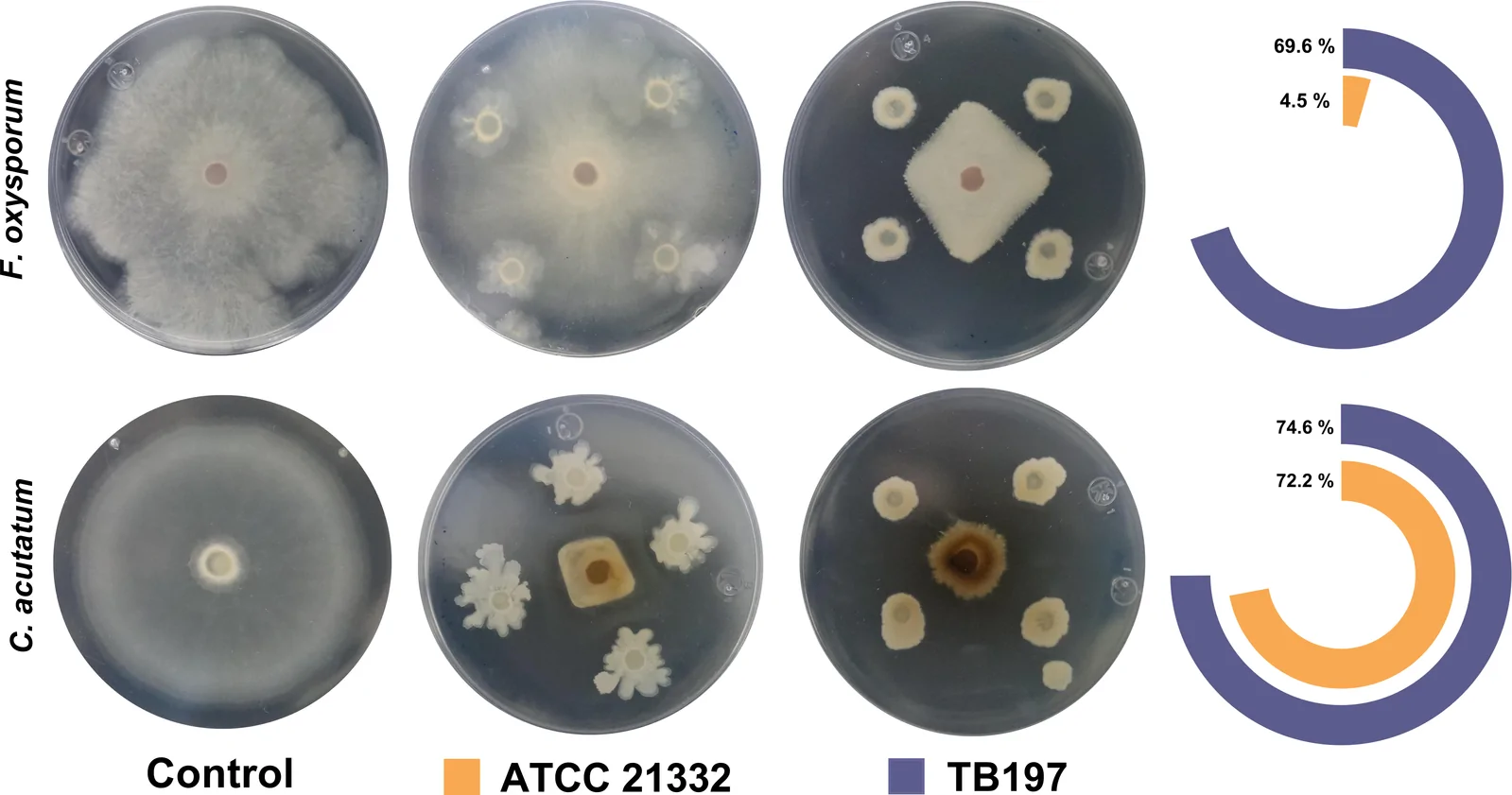
Antifungal activity of *B. subtilis* ATCC 21332 and *B. paralicheniformis* TB197 against *F. oxysporum* and *C. acutatum*. Circular bars represent the percentage of mycelial growth inhibition produced by each bacterial strain. Orange bars correspond to ATCC 21332, whereas purple bars correspond to B. TB197.

The TB197 strain showed substantial *in vitro* antifungal activity against *F. oxysporum* and *C. acutatum* (Fig. 6). For *F. oxysporum*, the inhibition was 15.5 times significantly higher (*P* ≤ 0.05) than that of the reference strain (ATCC 21332). Against *C. acutatum*, both strains exhibited strong inhibition (*P* ≤ 0.05), with rates of approximately 74%. Genomically, TB197 contains genes associated with the production of secondary metabolites with known antifungal effects, particularly non-ribosomal peptide synthetases involved in the synthesis of surfactin and fengycin (Table 1), both of which are widely recognized for their antifungal properties (82).

#### Nematicidal activities

The strain TB197 has previously been shown to exhibit a remarkable capacity to control plant-parasitic nematodes (19), a capacity subsequently confirmed to result from the synthesis of a diverse array of cyclic lipopeptides (fengycins, surfactins, and lichenysins). Among these metabolites, the fengycin family displayed even higher activity in semi-purified fractions (83). Further studies focused on culture conditions that optimize lipopeptide biosynthesis confirmed the expression of non-ribosomal peptide synthetase (NRPS) genes, including *fen*B and *lich*A, which are responsible for biosynthesis of fengicins, surfactins, and lichenysins (53). The high efficacy of TB197 against plant-parasitic nematodes, coupled with its adaptability to diverse environments and agricultural systems, motivated the present study, which aimed to elucidate its agrobiotechnological potential.

#### Extracellular enzymes

Using the dbCAN3 tool, 168 enzymatic domains were identified in the genome of the TB197 strain. Among these, 72 were glycoside hydrolases (GHs), 45 glycosyltransferases (GTs), 21 carbohydrate esterases (CEs), 15 carbohydrate-binding modules (CBMs), 8 polysaccharide lyases (PLs), and 4 auxiliary activity enzymes (AAs) (Table S1). From this data set, we specifically filtered extracellular enzymes involved in degrading plant cell-wall-relevant polysaccharide residues, such as cellulose (cellulases), xylan (xylanases), starch (amylases), and pectin (pectinases), because of their roles in nutrient mobilization; besides others like chitin (chitinases), which are involved in antimicrobial activity, especially against phytopathogenic fungi (82, 84). A total of 16 cellulases, 13 amylases, 10 chitinases, 7 pectinases, and 5 extracellular xylanases were identified (Fig. S1). Additionally, a semi-quantitative assay was performed to assess the cellulase activity of the TB197 strain *in vitro*. Results showed detectable cellulase activity, although it was 3.3 times lower than that of the ATCC 21332 strain used as a positive control (Fig. S3). These results indicate that the TB197 strain possesses a wide range of extracellular enzymes involved in the degradation of plant polysaccharides, suggesting its potential for nutrient mobilization and interaction with the soil microbiome. However, more studies are needed to understand how these enzymes are regulated and how active they are under rhizosphere-like conditions.

### Conclusions

Initially classified as a BCA bacterium highly effective in controlling phytopathogenic nematodes, whole-genome sequencing and genome mining reveal that the *B. paralicheniformis* TB197 strain has an extensive, metabolically active genetic repertoire directly linked to promoting plant growth and effectively controlling phytopathogens beyond nematodes.

The genome mining approach revealed a much broader metabolic potential than traditional phenotype-based methods. Genes and *in vitro* activities related to the synthesis of phytohormones, volatile organic compounds (VOCs), siderophores, root colonization ability, even under extreme conditions like high temperatures and salinity, along with improvements in nutrient and water uptake by plants, clearly identify the TB197 strain as an excellent microbial biostimulant for standard or regenerative agricultural practices. Additionally, the identification of numerous genes associated with plant abiotic stress mitigation (e.g., salinity, temperature, and heavy metals) provides a strong genetic basis for future studies to experimentally validate the strain’s effectiveness in protecting crops under field conditions and to assess its potential applications in bioremediation.

Finally, the presence of genes encoding antimicrobial metabolites, including nematicidal, bactericidal (mainly targeting Gram-positive bacteria), and fungicidal compounds, along with enzymes that target phytopathogenic fungal cell walls, highlights the biotechnological potential of the TB197 strain as a strong candidate for biological control of various plant pathogens in agriculture. Furthermore, uncharacterized biosynthetic pathways suggest the existence of new bioactive compounds with significant biotechnological potential, including novel antimicrobial agents. This makes the TB197 strain a valuable resource for future research and industrial applications.

## Data Availability

The corresponding author will provide data sets generated and/or analyzed during this study upon reasonable request.

## SUPPLEMENTAL MATERIAL

Supplemental figures and tables spectrum.00441-26-s0001.docx Figures S1 to S3 and Tables S1 and S2.

ASM does not own the copyrights to Supplemental Material that may be linked to, or accessed through, an article. The authors have granted ASM a non-exclusive, world-wide license to publish the Supplemental Material files. Please contact the corresponding author directly for reuse.
