# Supplementary material for: Genomic exploration of Bacillus paralicheniformis TB197: an agrobiotechnological tool from the Sonoran Desert
Source: Microbiol Spectr. 2026 Jul 22;14(9):e00441-26. doi: 10.1128/spectrum.00441-26 (PMC13532188; doi:10.1128/spectrum.00441-26)
Supplement: Supplementary file 1 [file spectrum.00441-26-s0001.docx]

**Supplementary information**

**Genomic Exploration of Bacillus paralicheniformis TB197: An Agrobiotechnological Tool from the Sonoran Desert**

Estefany Chavarria-Quicaño^1^, Miguel A. Hernández-Oñate^2^, Jorge D. Payan-Almanza^1^, Viridiana Rubio-Maldonado^1^, Francisco de la Torre-González^1^, and Ali Asaff-Torres^3*^

**Supplementary Figure 1.** Examples of photographs used to calculate the leaf area of lettuce plants

**Supplementary Figure 2.** Relative antibacterial activity of *Bacillus paralicheniformis* TB197 with respect to *Bacillus subtilis* ATCC 21332.

**Supplementary Figure 3.** Ex Semiquantitative production of cellulases. On the left *B. subtilis* ATCC 21332; On the right *B. paralicheniformis* TB197 strain; c) Cellulase production index.

**Supplementary table 1.** Enzymatic domains identified in *Bacillus paralicheniformis* TB197 strain using the dbCAN3 tool.

**Gene ID	EC#	HMMER	dbCAN_sub	DIAMOND	Signalp #ofTools**

Bacillus_1017	-	CE4(100-228)	-	-	N	1

Bacillus_1070	-	GT51(52-226)	-	-	N	1

Bacillus_1123	-	GH4(3-181)	-	-	N	1

Bacillus_1163	-	GH188(5-228)	-	-	N	1

Bacillus_1229	-	GH8(83-424)	-	-	Y(1-29) 1

Bacillus_1241	-	GH23(112-215)	-	-	N	1

Bacillus_1275	-	CE1(15-237)	-	-	N	1

Bacillus_1286	-	GH43_5(36-314) -	-	Y(1-26) 1

Bacillus_13	-	GH18(167-416)	-	-	N	1

Bacillus_1329	-	CBM50(161-218) -	-	N	1

Bacillus_1358	-	GH105(25-336)	-	-	N	1

Bacillus_1360	-	CE12(12-216)	-	-	N	1

Bacillus_1363	-	PL11(40-621)	-	-	Y(1-33) 1

Bacillus_1364	-	CE12(321-529)	-	-	Y(1-32) 1

Bacillus_1365	-	PL11(23-622)	-	-	N	1

Bacillus_1367	-	CE12(5-199)	-	-	N	1

Bacillus_1368	-	GH42(12-373)	-	-	N	1

Bacillus_1369	-	PL26(6-841)	-	-	N	1

Bacillus_1390	-	PL1_6(136-345) -	-	N	1

Bacillus_154	-	CE4(51-179)	-	-	N	1

Bacillus_1580	-	GT119(17-372)	-	-	N	1

Bacillus_161	-	GH171(52-409)	-	-	Y(1-23) 1

Bacillus_1616	-	GT119(1-337)	-	-	N	1

Bacillus_1617	-	GT28(188-350)	-	-	N	1

Bacillus_162	-	GH3(115-349)	-	-	Y(1-29) 1

Bacillus_1754	-	GH9(41-475)+CBM3(501-586)	-	-	Y(1-37) 1

Bacillus_1755	-	GH48(4-463)	-	-	N	1

Bacillus_1756	-	GH5_4(71-331)+CBM46(368-447)	-	-	Y(1-34) 1

Bacillus_1757	-	GH5_7(66-340)	-	-	Y(1-32) 1

Bacillus_1769	-	CE4(125-247)	-	-	N	1

Bacillus_179	-	GH1(8-475)	-	-	N	1

Bacillus_1827	-	GT1(171-391)	-	-	N	1

Bacillus_1831	-	GH18(181-403)	-	-	N	1

Bacillus_186	-	CE7(5-314)	-	-	N	1

Bacillus_1925	-	AA6(21-211)	-	-	N	1

Bacillus_1985	-	GH5_2(54-292)+CBM3(375-455)	-	-	Y(1-33) 1

Bacillus_1992	-	PL9_2(32-390)	-	-	Y(1-22) 1

Bacillus_2015	-	GH31_2(245-690) -	-	N	1

Bacillus_2030	-	GT4(212-366)	-	-	N	1

Bacillus_2067	-	GT2(9-179)	-	-	N	1

Bacillus_2117	-	GT4(222-371)	-	-	N	1

Bacillus_2186	-	CE14(6-124)	-	-	N	1

Bacillus_2191	-	CBM63(139-214) -	-	Y(1-26) 1

Bacillus_2251	-	GT28(205-353)	-	-	N	1

Bacillus_226	-	GH18(68-521)	-	-	Y(1-27) 1

Bacillus_227	-	GH18(37-434)	-	-	Y(1-33) 1

Bacillus_2292	-	GT51(86-261)	-	-	N	1

Bacillus_230	-	GH4(2-153)	-	-	N	1

Bacillus_2306	-	GT4(197-345)	-	-	N	1

Bacillus_2307	-	CE14(5-110)	-	-	N	1

Bacillus_234	-	AA4(12-237)	-	-	N	1

Bacillus_2372	-	CE4(75-179)	-	-	N	1

Bacillus_2485	-	GH1(12-470)	-	-	N	1

Bacillus_2588	-	GT4(190-336)	-	-	N	1

Bacillus_2690	-	CE12(26-205)	-	-	Y(1-22) 1

Bacillus_2705	-	GH4(6-186)	-	-	N	1

Bacillus_2774	-	GH43_11(3-301)+CBM91(329-532)	-	-	N	1

Bacillus_2778	-	GH32(39-349)+CBM66(520-673)	-	-	Y(1-25) 1

Bacillus_2972	-	GH51_1(8-498)	-	-	N	1

Bacillus_2980	-	GH43_5(40-318) -	-	Y(1-30) 1

Bacillus_3003	-	PL1_8(221-417) -	-	Y(1-31) 1

Bacillus_3013	-	GH16_21(37-238) -	-	Y(1-30) 1

Bacillus_3089	-	CBM68(6-98)+GH13_14(261-565)	-	-	N	1

Bacillus_3110	-	GH188(28-211)	-	-	N	1

Bacillus_3112	-	GH105(30-363)	-	-	N	1

Bacillus_3118	-	CE19(10-290)	-	-	N	1

Bacillus_3141	-	GT4(196-342)	-	-	N	1

Bacillus_3175	-	GT35(93-795)	-	-	N	1

Bacillus_3176	-	GT5(2-473)	-	-	N	1

Bacillus_3179	-	CBM48(26-108)+GH13_9(177-467)	-	-	N	1

Bacillus_3214	-	GT28(201-344)	-	-	N	1

Bacillus_3219	-	GH188(2-145)	-	-	N	1

Bacillus_3228	-	GH13_31(28-267) -	-	N	1

Bacillus_3231	-	GH12(108-260)	-	-	Y(1-30) 1

Bacillus_3255	-	GT51(49-224)	-	-	N	1

Bacillus_3415	-	CE8(11-310)	-	-	N	1

Bacillus_3420	-	GH33(38-372)	-	-	N	1

Bacillus_3431	-	GH28(51-407)	-	-	N	1

Bacillus_3462	-	GH31_3(237-665) -	-	N	1

Bacillus_3463	-	GH3(73-304)+CBM92(460-573)+CBM6(862-980)	-	-	Y(1-30) 1

Bacillus_3490	-	GH179(39-195)	-	-	N	1

Bacillus_3500	-	GH30_8(30-416) -	-	Y(1-26) 1

Bacillus_3501	-	GH43_16(38-360)+CBM6(382-511)	-	-	Y(1-27) 1

Bacillus_3549	-	GT2(6-160)	-	-	N	1

Bacillus_3551	-	GT2(7-170)	-	-	N	1

Bacillus_3552	-	GT122(40-352)	-	-	N	1

Bacillus_3553	-	GT4(204-314)	-	-	N	1

Bacillus_3554	-	GT2(10-165)	-	-	N	1

Bacillus_3555	-	GT4(191-344)	-	-	N	1

Bacillus_3571	-	GH68_1(39-478) -	-	Y(1-30) 1

Bacillus_3572	-	GH32(26-327)	-	-	N	1

Bacillus_3603	-	PL3_1(28-204)	-	-	Y(1-29) 1

Bacillus_3660	-	GT2(9-138)	-	-	N	1

Bacillus_3665	-	GT4(214-356)	-	-	N	1

Bacillus_3676	-	GT2(11-171)	-	-	N	1

Bacillus_3677	-	GT2(3-166)	-	-	N	1

Bacillus_3682	-	GT26(58-225)	-	-	N	1

Bacillus_3684	-	GH73(731-858)	-	-	Y(1-29) 1

Bacillus_3772	-	CE1(30-263)	-	-	N	1

Bacillus_380	-	CE1(24-257)	-	-	N	1

Bacillus_3811	-	GH188(2-221)	-	-	N	1

Bacillus_3843	-	GT51(62-238)	-	-	Y(1-41) 1

Bacillus_3877	-	GT2(4-163)	-	-	N	1

Bacillus_3880	-	GH32(32-337)	-	-	N	1

Bacillus_3890	-	GT119(21-379)	-	-	N	1

Bacillus_3891	-	GT119(19-383)	-	-	N	1

Bacillus_3928	-	GT8(4-257)	-	-	N	1

Bacillus_3951	-	GH4(6-185)	-	-	N	1

Bacillus_3965	-	CE12(7-210)	-	-	N	1

Bacillus_3981	-	PL1_5(100-274) -	-	Y(1-22) 1

Bacillus_4002	-	CE4(115-241)	-	-	Y(1-25) 1

Bacillus_4005	-	GT2(106-276)	-	-	N	1

Bacillus_4019	-	GH1(7-469)	-	-	N	1

Bacillus_4025	-	GH32(27-330)	-	-	N	1

Bacillus_4034	-	GT83(9-261)	-	-	N	1

Bacillus_4035	-	GT2(15-177)	-	-	N	1

Bacillus_4044	-	GH1(7-468)	-	-	N	1

Bacillus_4055	-	GT83(12-230)	-	-	N	1

Bacillus_4069	-	GH1(5-466)	-	-	N	1

Bacillus_4075	-	GH43_4(39-360) -	-	Y(1-29) 1

Bacillus_4087	-	GH126(34-342)	-	-	N	1

Bacillus_4090	-	GT2(49-279)	-	-	N	1

Bacillus_4100	-	GH188(3-159)	-	-	N	1

Bacillus_4131	-	GH53(50-413)	-	-	Y(1-27) 1

Bacillus_4132	-	GH42(17-396)	-	-	N	1

Bacillus_418	-	GH42(20-394)	-	-	N	1

Bacillus_4184	-	CE9(27-405)	-	-	N	1

Bacillus_496	-	AA10(32-199)	-	-	Y(1-32) 1

Bacillus_502	-	GT1(157-391)	-	-	N	1

Bacillus_546	-	GH11(32-211)	-	-	Y(1-29) 1

Bacillus_559	-	GH177(34-286)	-	-	N	1

Bacillus_626	-	GT119(16-372)	-	-	N	1

Bacillus_627	-	GT119(24-368)	-	-	N	1

Bacillus_631	-	CE4(267-380)	-	-	N	1

Bacillus_632	-	GH13_5(103-440) -	-	N	1

Bacillus_634	-	CBM34(6-127)+GH13_20(176-469)	-	-	N	1

Bacillus_639	-	GH65(323-693)	-	-	N	1

Bacillus_640	-	GH13_31(27-377) -	-	N	1

Bacillus_654	-	AA1(48-498)	-	-	N	1

Bacillus_710	-	GH26(30-347)	-	-	Y(1-25) 1

Bacillus_720	-	GH1(6-485)	-	-	N	1

Bacillus_768	-	GH18(30-331)	-	-	Y(1-28) 1

Bacillus_780	-	GH13_29(30-376) -	-	N	1

Bacillus_791	-	GT2(49-151)	-	-	N	1

Bacillus_792	-	GT4(235-352)	-	-	N	1

Bacillus_793	-	GT2(5-143)	-	-	N	1

Bacillus_795	-	GH188(4-201)	-	-	N	1

Bacillus_814	-	CE4(60-186)	-	-	Y(1-25) 1

Bacillus_827	-	GT1(184-391)	-	-	N	1

Bacillus_844	-	GH4(7-184)	-	-	N	1

Bacillus_852	-	GH43_10(26-293)+CBM91(328-523) -	-	N	1

Bacillus_853	-	GH43_12(8-289)+CBM91(316-513)	-	-	N	1

**Supplementary Table 2. List of extracellular CAZymes**

| **Family** | **Activity Id** | **EC#** | **Activity Name** |
| --- | --- | --- | --- |
| **Cellulases** | | | |
| **Glycoside hydrolase families 5/8/9/12** | H4 | 3.2.1.4 | cellulose endo-β-1,4-glucosidase |
|  | H5 | 3.2.1.6 | MLG endo-β-1,3/4-glucosidase |
|  | H7 | 3.2.1.8 | xylan endo-b-1,4-xylosidase |
|  | H19 | 3.2.1.25 | β-mannosidase |
|  | H23 | 3.2.1.32 | xylan endo-β-1,3-xylosidase |
|  | H55 | 3.2.1.73 | MLG endo-β-1,4-glucosidase |
|  | H106 | 3.2.1.132 | chitosan endo-β-1,4-N-glucosaminase |
|  | H122 | 3.2.1.156 | reducing end xylan exo-β-1,4-xylosidase |
|  | H56 | 3.2.1.74 | cellulose exo-β-1,4-glucosidase |
|  | H59 | 3.2.1.78 | mannan endo-β-1,4-mannosidase |
|  | H68 | 3.2.1.91 | cellulose exo-β-1,4-glucobiosidase |
|  | H118 | 3.2.1.151 | xyloglucan endo-β-1,4-glucosidase |
|  | H130 | 3.2.1.165 | chitosan exo-β-1,4-N-glucosaminase |
|  | H226 | 3.2.1.- | xanthan endo-β-1,4-glucosidase |
|  | H192 | 3.2.1.- | MLG endo-β-1,3-glucosidase |
|  | H228 | 3.2.1.- | glucomannan endo-β-1,4-glucosidase |
| **Xylanases** | | | |
| **Glycoside Hydrolase Family**  **43_4**  **43_5**  **43_7**  **43_10**  **43_12**  **43_16** | H7 | 3.2.1.8 | xylan endo-β-1,4-xylosidase |
|  | H27 | 3.2.1.37 | xylan exo-β-1,4-xylosidase |
|  | H41 | 3.2.1.55 | α-L-arabinofuranosidase |
|  | H54 | 3.2.1.72 | xylan exo-β-1,3-xylosidase |
|  | H76 | 3.2.1.99 | arabinan endo-α-1,5-arabinofuranosidase |
|  | H180 | 3.2.1 | arabinan exo-α-1,5-L-arabinofuranosidase |
| **Pectinases** | | | |
| **Carbohydrate esterase Family 12** | CE12 | 3.1.1 | Carboxylic ester hydrolase |
|  | CE12 | 3.1.1 | Pectin acetylesterase |
|  | CE12 | 3.1.1.72 | Acetylxylan esterase |
|  | CE12 | 3.1.1.86 | Rhamnogalacturonan acetylesterase |
| **Polysaccharide Lyase Family**  **1_6**  **1_5**  **9_2** | L357 | 4.2.2.2 | pectate lyase |
|  | L363 | 4.2.2.9 | exo-unsaturated disaccharide pectate lyase |
|  | L364 | 4.2.2.10 | pectin lyase |
|  |  | 4.2.2.2 | pectate lyase |
|  |  | 4.2.2.23 | Rhamnogalacturonan endolyase |
|  |  | 4.2.2. | Thiopeptidoglycan lyase |
|  |  | 4.2.2.9 | Exo-polygalacturonate lyase |
| **Carbohydrate Esterase family 8** |  | 3.1.1.11 | Pectin methylesterase |
| **Glucoside Hydrolase family 28** | H11 | 3.2.1.15 | galacturonan endo-α-1,4-galacturonidase |
|  | H50 | 3.2.1.67 | galacturonan exo-α-1,4-galacturonidase |
|  | H61 | 3.2.1.82 | galacturonan exo-α-1,4-galacturobiodase |
|  | H136 | 3.2.1.171 | rhamnogalacturonan I endo-α-1,2-galacturonidase |
|  | H138 | 3.2.1.173 | rhamnogalacturonan I exo-α-1,2-galacturonidase |
|  | H177 | 3.2.1 | xylogalacturonan endo-α-1,4-galacturonidase |
| **Amilases** | | | |
| **Glycoside Hydrolase Family**  **13_5**  **13_11**  **13_29**  **13_31** | H1 | 3.2.1.1 | amylose endo-α-1,4-glucosidase |
|  | H3 | 3.2.1.3 | amylose exo-α-1,4-glucosidase |
|  | H8 | 3.2.1.10 | oligo exo-α-1,6-glucosidase |
|  | H14 | 3.2.1.20 | α-glucosidase |
|  | H52 | 3.2.1.70 | dextran exo-α-1,6-glucosidase |
|  | H70 | 3.2.1.93 | phosphotrehalose α-1,1-(6 phospho)-glucosidase |
|  | H75 | 3.2.1.98 | amylose exo-α-1,4-glucohexaosidase |
|  | H91 | 3.2.1.116 | amylose exo-α-1,4-glucotriosidase |
|  | H182 | 3.2.1 | amylose exo-1,4-glucopentaosidase |
|  | H191 | 3.2.1 | palatin α-1,6-glucosidase |
|  | T318 | 2.4.1.161 | oligosaccharide 4-α-D-glucosyltransferase |
|  | T342 | 2.4.1 | sucrose α-transglucosidase |
|  | M348 | 5.4.99.11 | isomaltulose synthase |
| **Chitinases** | | | |
| **Glycoside Hydrolase Family 18** | H10 | 3.2.1.14 | chitin endo-β-1,4-N-acetylglucosaminidase |
|  | H38 | 3.2.1.52 | β-N-acetylhexosaminidase |
|  | H73 | 3.2.1.96 | mannosyl-oligosaccharide endo-β-1,4-N-acetylglucosaminidase |
|  | H106 | 3.2.1.132 | chitosan endo-β-1,4-N-glucosaminase |
|  | H155 | 3.2.1.200 | chitin exo-β-1,4-N-acetylglucobiosaminidase |
|  | H156 | 3.2.1.201 | reducing end chitin exo-β-1,4-N-acetylglucosaminidase |
|  | H175 | 3.2.1 | mannosyl-oligosaccharide exo-β-1,4-N-acetylglucosaminidase |
|  | H181 | 3.2.1 | chitin exo-β-1,4-N-acetylglucosaminidase |
|  | H183 | 3.2.1 | peptidoglycan endo-βα-1,4-N-acetylglucosaminidase |
|  | H205 | 3.2.1 | Nod factor endo-β-1,4-N-acetylglucosaminidase |

| **Family** | **Activity Id** | **EC#** | **Activity Name** |
| --- | --- | --- | --- |
| **Cellulases** | | | |
| **Glycoside hydrolase families 5/8/9/12** | H4 | 3.2.1.4 | cellulose endo-β-1,4-glucosidase |
|  | H5 | 3.2.1.6 | MLG endo-β-1,3/4-glucosidase |
|  | H7 | 3.2.1.8 | xylan endo-b-1,4-xylosidase |
|  | H19 | 3.2.1.25 | β-mannosidase |
|  | H23 | 3.2.1.32 | xylan endo-β-1,3-xylosidase |
|  | H55 | 3.2.1.73 | MLG endo-β-1,4-glucosidase |
|  | H106 | 3.2.1.132 | chitosan endo-β-1,4-N-glucosaminase |
|  | H122 | 3.2.1.156 | reducing end xylan exo-β-1,4-xylosidase |
|  | H56 | 3.2.1.74 | cellulose exo-β-1,4-glucosidase |
|  | H59 | 3.2.1.78 | mannan endo-β-1,4-mannosidase |
|  | H68 | 3.2.1.91 | cellulose exo-β-1,4-glucobiosidase |
|  | H118 | 3.2.1.151 | xyloglucan endo-β-1,4-glucosidase |
|  | H130 | 3.2.1.165 | chitosan exo-β-1,4-N-glucosaminase |
|  | H226 | 3.2.1.- | xanthan endo-β-1,4-glucosidase |
|  | H192 | 3.2.1.- | MLG endo-β-1,3-glucosidase |
|  | H228 | 3.2.1.- | glucomannan endo-β-1,4-glucosidase |
| **Xylanases** | | | |
| **Glycoside Hydrolase Family**  **43_4**  **43_5**  **43_7**  **43_10**  **43_12**  **43_16** | H7 | 3.2.1.8 | xylan endo-β-1,4-xylosidase |
|  | H27 | 3.2.1.37 | xylan exo-β-1,4-xylosidase |
|  | H41 | 3.2.1.55 | α-L-arabinofuranosidase |
|  | H54 | 3.2.1.72 | xylan exo-β-1,3-xylosidase |
|  | H76 | 3.2.1.99 | arabinan endo-α-1,5-arabinofuranosidase |
|  | H180 | 3.2.1 | arabinan exo-α-1,5-L-arabinofuranosidase |
| **Pectinases** | | | |
| **Carbohydrate esterase Family 12** | CE12 | 3.1.1 | Carboxylic ester hydrolase |
|  | CE12 | 3.1.1 | Pectin acetylesterase |
|  | CE12 | 3.1.1.72 | Acetylxylan esterase |
|  | CE12 | 3.1.1.86 | Rhamnogalacturonan acetylesterase |
| **Polysaccharide Lyase Family**  **1_6**  **1_5**  **9_2** | L357 | 4.2.2.2 | pectate lyase |
|  | L363 | 4.2.2.9 | exo-unsaturated disaccharide pectate lyase |
|  | L364 | 4.2.2.10 | pectin lyase |
|  |  | 4.2.2.2 | pectate lyase |
|  |  | 4.2.2.23 | Rhamnogalacturonan endolyase |
|  |  | 4.2.2. | Thiopeptidoglycan lyase |
|  |  | 4.2.2.9 | Exo-polygalacturonate lyase |
| **Carbohydrate Esterase family 8** |  | 3.1.1.11 | Pectin methylesterase |
| **Glucoside Hydrolase family 28** | H11 | 3.2.1.15 | galacturonan endo-α-1,4-galacturonidase |
|  | H50 | 3.2.1.67 | galacturonan exo-α-1,4-galacturonidase |
|  | H61 | 3.2.1.82 | galacturonan exo-α-1,4-galacturobiodase |
|  | H136 | 3.2.1.171 | rhamnogalacturonan I endo-α-1,2-galacturonidase |
|  | H138 | 3.2.1.173 | rhamnogalacturonan I exo-α-1,2-galacturonidase |
|  | H177 | 3.2.1 | xylogalacturonan endo-α-1,4-galacturonidase |
| **Amilases** | | | |
| **Glycoside Hydrolase Family**  **13_5**  **13_11**  **13_29**  **13_31** | H1 | 3.2.1.1 | amylose endo-α-1,4-glucosidase |
|  | H3 | 3.2.1.3 | amylose exo-α-1,4-glucosidase |
|  | H8 | 3.2.1.10 | oligo exo-α-1,6-glucosidase |
|  | H14 | 3.2.1.20 | α-glucosidase |
|  | H52 | 3.2.1.70 | dextran exo-α-1,6-glucosidase |
|  | H70 | 3.2.1.93 | phosphotrehalose α-1,1-(6 phospho)-glucosidase |
|  | H75 | 3.2.1.98 | amylose exo-α-1,4-glucohexaosidase |
|  | H91 | 3.2.1.116 | amylose exo-α-1,4-glucotriosidase |
|  | H182 | 3.2.1 | amylose exo-1,4-glucopentaosidase |
|  | H191 | 3.2.1 | palatin α-1,6-glucosidase |
|  | T318 | 2.4.1.161 | oligosaccharide 4-α-D-glucosyltransferase |
|  | T342 | 2.4.1 | sucrose α-transglucosidase |
|  | M348 | 5.4.99.11 | isomaltulose synthase |
| **Chitinases** | | | |
| **Glycoside Hydrolase Family 18** | H10 | 3.2.1.14 | chitin endo-β-1,4-N-acetylglucosaminidase |
|  | H38 | 3.2.1.52 | β-N-acetylhexosaminidase |
|  | H73 | 3.2.1.96 | mannosyl-oligosaccharide endo-β-1,4-N-acetylglucosaminidase |
|  | H106 | 3.2.1.132 | chitosan endo-β-1,4-N-glucosaminase |
|  | H155 | 3.2.1.200 | chitin exo-β-1,4-N-acetylglucobiosaminidase |
|  | H156 | 3.2.1.201 | reducing end chitin exo-β-1,4-N-acetylglucosaminidase |
|  | H175 | 3.2.1 | mannosyl-oligosaccharide exo-β-1,4-N-acetylglucosaminidase |
|  | H181 | 3.2.1 | chitin exo-β-1,4-N-acetylglucosaminidase |
|  | H183 | 3.2.1 | peptidoglycan endo-βα-1,4-N-acetylglucosaminidase |
|  | H205 | 3.2.1 | Nod factor endo-β-1,4-N-acetylglucosaminidase |
